# Temporal trends in chronic diseases among offshore oil workers and the interaction effect of age with body mass index

**DOI:** 10.3389/fpubh.2025.1738126

**Published:** 2025-12-10

**Authors:** Jinru Li, Mingqiu Hu, Tongyu Wang, Yanhao Xie, Yi Liu, Jian Yao, Guansen Hua, Xiangyu Yan, Haojun Fan

**Affiliations:** 1 School of Disaster and Emergency Medicine, Tianjin University, Tianjin, China; 2 The PRC Ministry of Education Engineering Research Center of Intelligent Technology for Healthcare, Wuxi, Jiangsu, China; 3 The Key Laboratory of Medical Rescue Key Technology and Equipment, Ministry of Emergency Management, Tianjin, China; 4 Tianjin PKUCare CNOOC Hospital, Tianjin, China; 5 School of Artificial Intelligence and Computer Science, Jiangnan University, Wuxi, China; 6 Xiamen Peiyang BCI & Smart Health Innovation Research Institution, Fujian, China

**Keywords:** occupational health, occupational population, offshore oil worker, interaction effect, risk factors

## Abstract

**Objective:**

To analyze trends in hypertension, diabetes, and dyslipidemia prevalence among Chinese offshore oil workers and explore the independent and interaction effects of age and BMI.

**Methods:**

Using health examination data of this population (2014–2024), we calculated the crude prevalence rate (CPR, the prevalence rate without age-structure adjustment) and the age-standardized prevalence rate (ASPR, adjusted to a standard population structure). Joinpoint regression assessed ASPR trends, and multivariable logistic regression analyzed age and BMI effects.

**Results:**

The overall mean CPR for hypertension, diabetes, and dyslipidemia from 2014 to 2024 were 22.41, 2.53, and 29.64%, respectively. Trend analysis revealed that ASPR for diabetes [Annual Percent Change (APC): 24.08, 95% CI: 12.93–35.23] and Dyslipidemia (APC: 21.83, 95% CI: 10.45–33.21) increased significantly (both *p* < 0.001), while hypertension trend was non-significant. In the risk factor analysis, both age (OR for hypertension = 1.03, 95% CI: 1.03–1.04; OR for diabetes = 1.11, 95% CI: 1.08–1.13; OR for Dyslipidemia = 1.02, 95% CI: 1.01–1.03) and BMI (OR for hypertension = 1.13, 95% CI: 1.11–1.15; OR for diabetes = 1.18, 95% CI: 1.12–1.22; OR for Dyslipidemia = 1.18, 95% CI: 1.16–1.20) were independent risk factors for all three conditions (all *p* < 0.001). A significant multiplicative interaction effect was observed among age and BMI, the group “Age >40 years and BMI ≥ 24 kg/m^2^” had the highest risk for hypertension (OR = 2.98, 95% CI: 2.37–3.74, *p* < 0.05), diabetes (OR = 16.11, 95% CI: 6.95–37.31), and Dyslipidemia (OR = 4.01, 95% CI: 3.30–4.88).

**Conclusion:**

The prevalence of chronic diseases among Chinese offshore oil workers is high, with diabetes and Dyslipidemia showing significant upward trends. Age and BMI are important influencing factors and exhibit an interaction effect. This population should be prioritized in occupational health surveillance, and comprehensive interventions focusing on weight management and metabolic screening should be implemented, particularly targeting middle-aged individuals with elevated BMI.

## Graphical abstract

**Figure:**
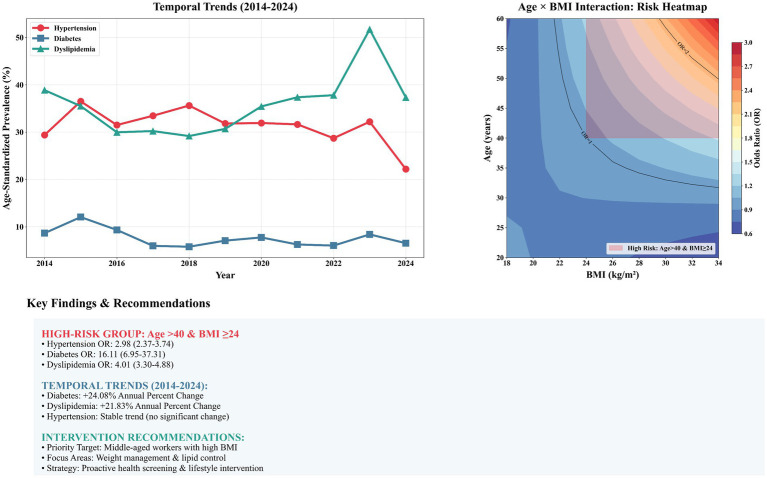

## Introduction

Offshore oil operations are classified by the International Labour Organization as a high-risk occupation, with occupational risks generally higher than most onshore work environments (1–6). This workforce is consistently exposed to a unique combination of work and living conditions, including shift work, confined living quarters, limited opportunities for physical activity, and a monotonous diet, all of which may elevate the risk of chronic diseases such as hypertension, diabetes, and Dyslipidemia (1, 3, 7–12). In recent years, with the expansion of offshore oil extraction and intensifying work rhythms, the health of this population has garnered increasing concern. International studies indicate that the prevalence of obesity, dyslipidemia, and abnormal glucose metabolism among offshore oil workers is generally higher than in onshore populations (1, 5, 10–12). For instance, the obesity rate among UK offshore workers was reported at 42.1% (13), and it was 37.8% among Deepwater Horizon oil spill cleanup workers (9, 14). However, these findings are predominantly derived from Western populations or combine offshore with onshore workers without disaggregation. Currently, systematic research on the chronic disease profile of Chinese offshore oil workers remains relatively scarce, and analyses of long-term trends, particularly the interaction effect of key influencing factors like age and body mass index (BMI), have not been reported (9, 15, 16).

China’s offshore oil industry is a crucial pillar of the national energy supply, with the Bohai Bay area contributing over 60% of the country’s total crude oil production (17). The number of employees on offshore platforms in this region is substantial, with the majority being medium- to long-term personnel. Their health status is directly linked to national energy security and the sustainable development of the occupational workforce. Although the China Occupational Health Protection Action Plan (2021–2025) emphasizes health surveillance for high-risk occupational groups (18), there is currently a lack of localized data on the epidemiological characteristics and influencing factors of chronic diseases among offshore oil workers, resulting in a lack of targeted prevention and control strategies. Against this backdrop, utilizing health examination data from employees working on offshore oil platforms in the Bohai Bay area from 2014 to 2024, this study systematically analyzed the current prevalence and long-term temporal trends of hypertension, diabetes, and Dyslipidemia. A key focus was placed on investigating the independent and interaction effects of age and BMI on the risk of these three chronic diseases. The findings aim to provide a scientific basis for developing early screening, risk stratification, and health management strategies tailored to this population, holding significant practical implications for advancing occupational health efforts in the offshore oil industry.

## Methods

### Study settings

This study was conducted among offshore oil platform workers in China’s Bohai Bay area, the nation’s primary offshore oil production region. The workforce operated on artificial island-style platforms under unique occupational conditions, following a 4-week on/off shift rotation. Workers were exposed to a distinct environment characterized by relative isolation, calorie-dense diets, and limited recreational options, which are considered potential risk factors for chronic metabolic diseases All offshore platform employees were required to undergo systematic annual health examinations at designated hospitals,

### Study design and population

This study utilized data from the occupational health monitoring system database of offshore oil platforms in the Bohai Bay area, covering the period from 2014 to 2024. The study population comprised employees who underwent annual health examinations at designated hospitals during this period. The study was reviewed and approved by the institutional review board of the Tianjin University Medical Ethics Committee (approval ID: TJUE-2024-434) and the Ethics Committee of PKU Care CNOOC Hospital. Informed consent was obtained from all participants prior to the study. This study had two main components: first, the analysis of long-term trends in the prevalence of hypertension, diabetes, and dyslipidemia from 2014 to 2024; and second, an in-depth investigation of the associated factors and their interactions, which primarily utilized the most recent cross-sectional data. Inclusion criteria were: (1) current employment on an offshore oil platform, (2) completion of an annual health check-up as part of the occupational health surveillance program, and (3) provision of written informed consent prior to participation. Exclusion criteria included: (1) temporary or short-term contract workers lacking systematic health monitoring records, and (2) individuals who declined to provide written informed consent during the health examination.

### Data collection variable definitions

Health examination data were systematically recorded in a centralized database. The dataset incorporated three categories of variables relevant to this analysis: (1) basic demographic information, including age and sex, (2) anthropometric measurements, specifically height and weight, which were used to calculate body mass index (BMI). Participants were categorized as underweight (BMI < 18.5), normal weight (18.5 ≤ BMI < 24), overweight (24 ≤ BMI < 28), or obese (BMI ≥ 28), and (3) clinical and laboratory indicators comprising systolic and diastolic blood pressure, fasting plasma glucose (GLU), total cholesterol (TCHO), low-density lipoprotein cholesterol (LDL-C), and triglycerides (TG). Glycated hemoglobin (HbA1c) was additionally measured for a subset of participants in 2024.

Diagnostic criteria for hypertension, diabetes, and Dyslipidemia were based on the Chinese Guidelines for the Prevention and Treatment of Hypertension (2024 Revision), the Chinese Guidelines for the Prevention and Treatment of Type 2 Diabetes (2020), and the Chinese Guidelines for the Prevention and Treatment of Dyslipidemia in Adults (2016 Revision), respectively (19–21). Hypertension was defined as systolic blood pressure ≥140 mmHg and/or diastolic blood pressure ≥90 mmHg, or a previous medical diagnosis. Diabetes was defined as fasting plasma glucose ≥7.0 mmol/L, or glycated hemoglobin (HbA1c) ≥ 6.5%, or a prior confirmed diagnosis. Dyslipidemia was defined as meeting any of the following criteria: total cholesterol (TC) ≥ 6.2 mmol/L, low-density lipoprotein cholesterol (LDL-C) ≥ 4.1 mmol/L, triglycerides (TG) ≥ 2.3 mmol/L, or a previous medical diagnosis. Overweight was defined as 24 kg/m^2^ ≤ BMI < 28 kg/m^2^, and obesity was defined as BMI ≥ 28 kg/m^2^. All physical examinations were conducted by qualified physicians and nurses, with key indicators verified independently by two professionals. To minimize potential bias in variable classification and disease diagnosis, all measurements and diagnoses were based on pre-defined clinical criteria as specified in the Chinese guidelines for hypertension, diabetes, and dyslipidemia (20–23).

### Statistical analysis

The analysis was structured in two phases to address the study’s objectives: chronic disease trend analysis and the investigation of risk factor associations and interactions. Data preparation included handling missing values through Multiple Imputation by Chained Equations (MICE). Subsequent models were fitted to the multiply imputed datasets, and results were pooled using Rubin’s rules to obtain final estimates. For the chronic disease trend analysis, we calculated two key metrics: the crude prevalence rate (CPR) and the age-standardized prevalence rate (ASPR). CPR is the prevalence rate calculated directly from the raw data, without any adjustment for age structure. To enable valid comparisons over time by removing the influence of changes in the population’s age structure, we calculated ASPR, which is the prevalence rate after adjusting for age structure, using the 2010 Chinese standard population composition (22). The Joinpoint Regression Program (Version 4.9.1.0) was employed to calculate the annual percent change (APC) and its 95% confidence interval (CI) for the ASPR, facilitating the analysis of long-term temporal trends.

To investigate the associations and interaction between age and BMI, the primary analysis was conducted using cross-sectional data from 2024, as it represents the most current health profile of the population, which is critical for informing contemporary occupational health interventions. To assess the temporal consistency and robustness of the findings, supplementary analyses were performed using data from the baseline year (2014) and a mid-point year (2019). Univariate logistic regression analyses were performed to assess the crude associations of age and body mass index (BMI) with each chronic disease. Both age and BMI demonstrated significant associations in these initial analyses. Multivariable logistic regression models were used to examine the associations of age and BMI with the prevalence of the three chronic diseases, with results expressed as odds ratios (OR) and their 95% CIs. All logistic regression models underwent Hosmer–Lemeshow goodness-of-fit tests and variance inflation factor (VIF) diagnostics to ensure adequate model fit and absence of severe multicollinearity (criterion: VIF < 5).

To assess the interaction effect of age and BMI, participants were categorized by age (using 40 years as the cutoff) and BMI (using 24 kg/m^2^ as the cutoff). An interaction effect analysis was performed, with the group “Age ≤40 years and BMI < 24 kg/m^2^” serving as the reference, and ORs were calculated for other combinations. To formally test the interaction effect between age and BMI, we further constructed multivariable logistic regression models incorporating an age × BMI interaction term. The significance of the interaction was assessed using the likelihood ratio test, comparing models with and without the interaction term. To account for multiple testing across the three chronic diseases, we applied Bonferroni correction for the interaction effect analyses, adjusting the significance threshold to *p* < 0.0056 (0.05/9 tests) for the nine interaction tests conducted (three diseases across three time points). All statistical analyses were performed using R software (Version 4.5.0) and Python (Version 3.12.3).

## Results

### Basic characteristics

This study was based on annual health examination data from employees working on offshore oil platforms in the Bohai Bay area, collected from designated hospitals between 2014 and 2024. A cumulative total of 4,815 participants were included in the analysis across the study years, with the overall monitored population size remaining stable. Demographically, the study population consisted predominantly of young and middle-aged men. The 31–40 years age group represented the highest proportion in most years; however, data from 2024 indicated a rising proportion of younger employees aged 20–30 years, which increased to 19.92%, suggesting a trend towards a younger age structure within this workforce. The distribution of body mass index (BMI) revealed that the combined prevalence of overweight and obesity exceeded 60%. Notably, the proportion of obesity increased from 13.37% in 2014 to 19.49% in 2024, indicating a growing concern regarding weight management in this population. All study subjects were incumbent employees of offshore oil platforms in the Bohai Bay area, ensuring good sample representativeness. The demographic characteristics of the study population were generally consistent across the different years, as detailed in Table 1.

**Table 1:** Characteristics of the study participants (2014–2024).

Demographic characteristics	2014	2015	2016	2017	2018	2019	2020	2021	2022	2023	2024
	*N* (%)	*N* (%)	*N* (%)	*N* (%)	*N* (%)	*N* (%)	*N* (%)	*N* (%)	*N* (%)	*N* (%)	*N* (%)
Total population (*N*)	3,544	3,801	3,788	3,792	3,825	3,874	3,984	4,252	4,304	4,466	4,241
Age group (years)											
20–30	5 (0.14)	10 (0.26)	10 (0.26)	10 (0.26)	32 (0.84)	64 (1.65)	172 (4.32)	344 (8.09)	668 (15.52)	848 (18.98)	845 (19.92)
31–40	1,852 (52.26)	2,123 (55.85)	2,108 (55.65)	2,110 (55.64)	2,128 (55.63)	2,147 (55.42)	2,147 (53.89)	2,200 (51.74)	2,128 (49.44)	2,137 (47.83)	1,989 (46.90)
41–50	1,252 (35.33)	1,246 (32.78)	1,244 (32.84)	1,243 (32.78)	1,242 (32.47)	1,236 (31.91)	1,243 (31.20)	1,275 (29.99)	1,136 (26.39)	1,122 (25.12)	1,058 (24.95)
51–61	435 (12.27)	422 (11.10)	426 (11.25)	429 (11.31)	423 (11.06)	427 (11.02)	422 (10.59)	433 (10.18)	372 (8.64)	359 (8.04)	349 (8.23)
BMI (kg/m^2^)											
Underweight (<18.5)	71 (2.00)	96 (2.53)	69 (1.82)	34 (0.90)	54 (1.41)	45 (1.16)	47 (1.18)	37 (0.87)	54 (1.25)	77 (1.72)	61 (1.44)
Normal (18.5 ≤ BMI < 23.9)	1,426 (40.24)	1,633 (42.96)	1,569 (41.42)	1,567 (41.32)	1,430 (37.39)	1,414 (36.50)	1,375 (34.51)	1,290 (30.34)	1,387 (32.23)	1,568 (35.11)	1,562 (36.83)
Overweight (24.0 ≤ BMI < 28.0)	1,573 (44.38)	1,479 (38.91)	1,674 (44.18)	1,716 (45.25)	1,808 (47.27)	1,831 (47.26)	1,858 (46.64)	1,912 (44.97)	2,039 (47.38)	1,841 (41.22)	1,791 (42.23)
Obese (≥28.0)	474 (13.38)	593 (15.60)	476 (12.58)	475 (12.53)	533 (13.94)	584 (15.07)	704 (17.67)	1,013 (23.82)	824 (19.15)	980 (21.94)	827 (19.50)

### Long-term trends in chronic disease prevalence

**Figure 1:**
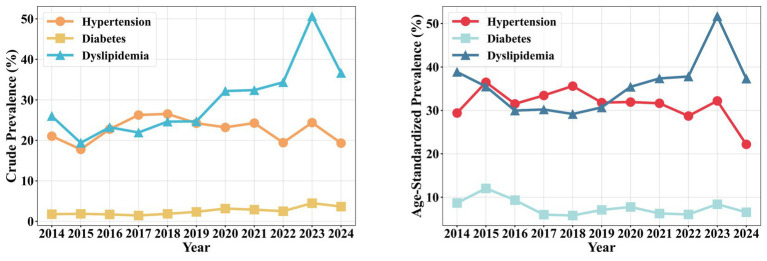
Chronic disease prevalence trends (2014–2024).

From 2014 to 2024, the overall mean CPR of hypertension, diabetes, and Dyslipidemia among the offshore oil workers was 22.41, 2.53, and 29.64%, respectively. Specifically, the crude prevalence of hypertension peaked in 2017 (26.27%), followed by a fluctuating decline to 19.34% in 2024. The crude prevalence of diabetes generally exhibited a fluctuating upward trend, reaching its lowest point in 2017 (1.45%) and rising to its highest level in 2023 (4.50%). The crude prevalence of Dyslipidemia showed considerable fluctuation, peaking in 2023 (50.65%) before decreasing to 36.64% in 2024; however, its age-standardized prevalence rate demonstrated a significant increasing trend during this period (APC = 21.83, 95% CI: 10.45–33.21, *p* = 0.002). It was noteworthy that in 2024, the CPR of both hypertension and Dyslipidemia showed a noticeable decline compared to 2023 (hypertension decreased from 24.38 to 19.34%; Dyslipidemia decreased from 50.65 to 36.64%). The results for ASPR from 2014 to 2024 indicated that while the rate for hypertension fluctuated, its overall trend was not statistically significant (APC = 0.54, 95% CI: −5.67–6.75, *p* = 0.986). In contrast, the ASPR for both diabetes (APC = 24.08, 95% CI: 12.93–35.23, *p* = 0.001) and Dyslipidemia (APC = 21.83, 95% CI: 10.45–33.21, *p* = 0.002) increased significantly (Figure 1). Furthermore, to better assess the actual burden of dysglycemia, HbA1c testing was performed on a subset of participants in 2024. The results revealed a pre-diabetes (HbA1c 5.7–6.4%) detection rate of 12.5%, and an additional 3.1% of workers had HbA1c ≥ 6.5% (already meeting the diagnostic criterion for diabetes). This indicated that the true burden of blood glucose metabolism abnormalities in this population is substantially higher than the prevalence rate (3.65%) derived solely from fasting blood glucose (see Table 2).

**Table 2:** Temporal trends of chronic disease prevalence (2014–2024).

Year	Hypertension			Diabetes			Dyslipidemia		
	Cases	CPR (%)	ASRPR (%)	Cases	CPR (%)	ASRPR (%)	Cases	CPR (%)	ASRPR (%)
2014	746	21.05	29.40	63	1.78	8.66	921	25.99	38.86
2015	676	17.78	36.50	71	1.87	12.05	737	19.39	35.50
2016	862	22.76	31.50	65	1.72	9.34	880	23.23	29.97
2017	996	26.27	33.46	55	1.45	5.97	832	21.94	30.22
2018	1,015	26.54	35.62	71	1.86	5.79	942	24.63	29.17
2019	939	24.24	31.81	91	2.35	7.08	957	24.70	30.69
2020	925	23.22	31.93	126	3.16	7.75	1,282	32.18	35.43
2021	1,032	24.27	31.64	123	2.89	6.26	1,378	32.41	37.37
2022	837	19.45	28.72	108	2.51	6.03	1,478	34.34	37.80
2023	1,089	24.38	32.20	201	4.50	8.39	2,262	50.65	51.70
2024	820	19.34	22.18	155	3.65	6.54	1,554	36.64	37.32

Prevalence is presented as crude prevalence (CPR, %) and age-standardized prevalence (ASPR, %). Cases are presented as number (*n*).

### Long-term prevalence trends in different subgroups

**Figure 2:**
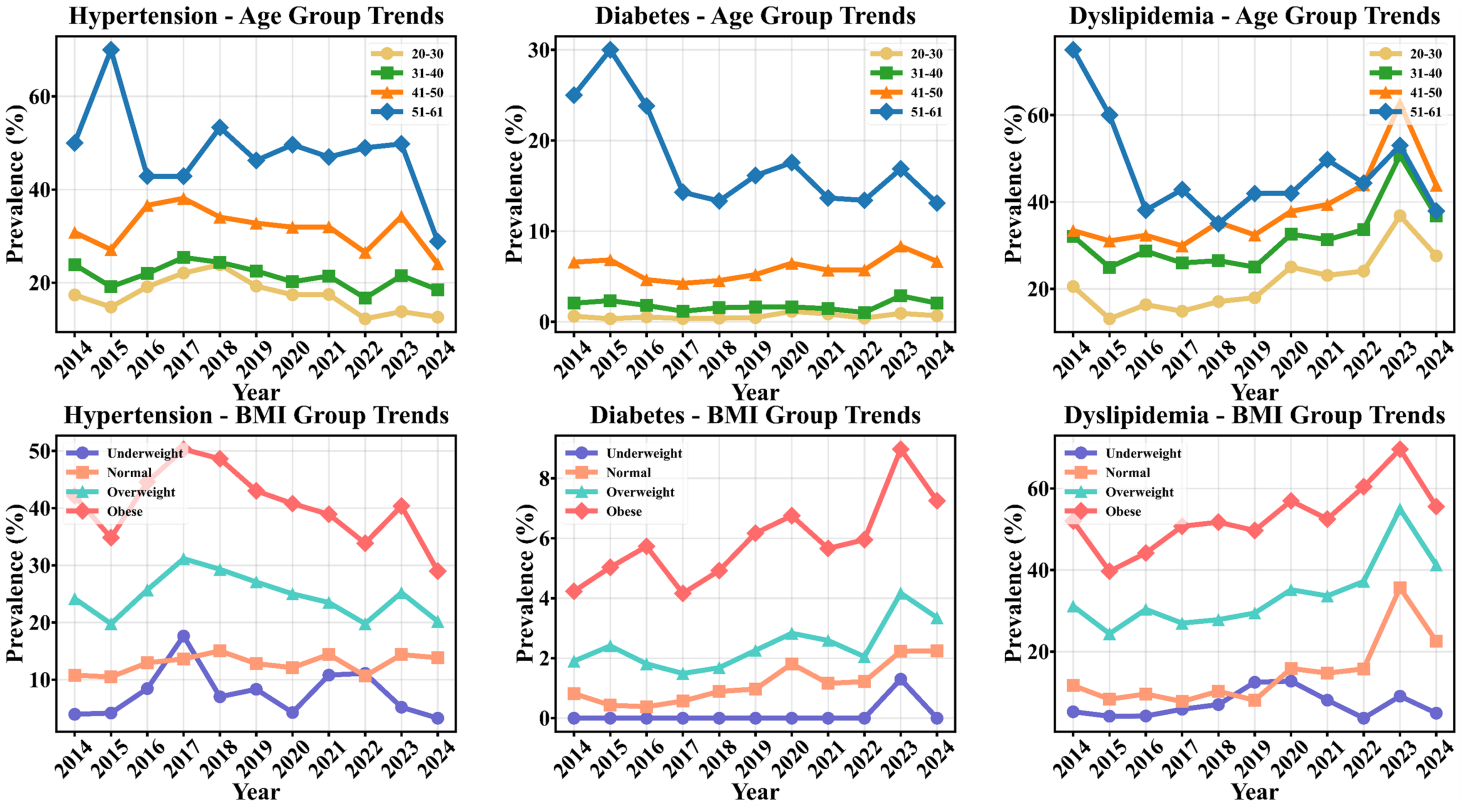
Chronic disease prevalence trends by subgroups (2014–2024).

From 2014 to 2024, the crude prevalence of chronic diseases exhibited distinct long-term trends across different age and BMI subgroups (Figure 2). Analysis by age group showed that hypertension prevalence decreased across all age groups, with the most pronounced reduction observed in the 51–61 years group. Diabetes prevalence showed a slight increase in the 20–30, 31–40, and 41–50 years groups, while it fluctuated and decreased in the 51–61 years group, peaking in 2015 (30.0%) before gradually declining. Dyslipidemia prevalence demonstrated a continuous increase in the 20–30, 31–40, and 41–50 years groups, with the most significant rise in the 41–50 years group. In contrast, the 51–61 years group showed no overall increase, with rates at relatively high levels in 2014–2015 followed by a decrease. Analysis by BMI subgroup revealed a slight decrease in hypertension prevalence among overweight and obese individuals, while a minor increase was observed in the normal-weight group. Diabetes prevalence increased across all BMI categories, with the most rapid rise in the obese group, reaching a peak of 9.10% in 2023. Dyslipidemia prevalence showed a consistent upward trend in the overweight, obese, and normal-weight groups, with the overweight and obese groups peaking at 54.80 and 69.00%, respectively, in 2023, while the underweight group showed no significant change. Notably, between 2023 and 2024, hypertension prevalence decreased markedly in several subgroups, particularly in the 51–61 years age group (from 32.10 to 28.90%) and among obese individuals (from 31.20 to 29.00%). During the same period, Dyslipidemia also showed a significant decline in the 51–61 years age group (from 45.20 to 37.90%), consistent with the overall downward trend, a phenomenon more evident in middle-aged and older individuals with higher BMI.

### Association factors and interaction effect analysis for chronic diseases

Based on the significant associations identified in univariate analyses, we proceeded to Multivable models to determine the independent and joint effects of age and BMI. Based on cross-sectional data from the most recent year (2024), with supplementary analyses from 2014 and 2019 for temporal comparison, the 51–61 years age group carried the most substantial burden of chronic diseases, with prevalence rates of hypertension, diabetes, and Dyslipidemia all marked higher than in other age groups; the 20–30 years group had the lowest prevalence rates. The distribution of cases across different combined age and BMI categories for the three chronic diseases was presented in Table 3. The majority of cases were concentrated in the 31–50 years age groups and among overweight and obese individuals (BMI ≥ 24.0 kg/m^2^). Specifically, hypertension and diabetes cases were more numerous in the overweight/obese subgroups of the 31–40 and 41–50 groups, while Dyslipidemia cases were particularly prominent in the overweight subgroup of the 31–40 years group (*n* = 360). Further analysis of prevalence distribution across subgroups revealed a clear increasing trend in the prevalence of all three chronic diseases with advancing age and increasing BMI. Even within the 20–30 years group, the prevalence of Dyslipidemia in overweight and obese individuals reached 42.00 and 33.30%, respectively, indicating that excess weight is a significant risk factor for metabolic abnormalities even in younger employees. In the 31–40 and 41–50 groups, hypertension prevalence exceeded 40% in both overweight and obese individuals, while diabetes prevalence was greater increased in obese subjects (51.20% in the 31–40 years group and 35.60% in the 41–50 years group). In the 51–61 years group, Dyslipidemia prevalence was highest in the overweight subgroup (54.90%), suggesting that the combination of older age and overweight represents a high-risk profile for dyslipidemia.

**Table 3:** Number of chronic disease cases by age and BMI groups.

Age group (years)	Hypertension				Diabetes				Dyslipidemia			
	Underweight	Normal	Overweight	Obese	Underweight	Normal	Overweight	Obese	Underweight	Normal	Overweight	Obese
20–30	0 (0.00%)	37 (33.33%)	37 (33.33%)	37 (33.33%)	0 (0.00%)	1 (16.67%)	2 (33.33%)	3 (50.00%)	1 (0.42%)	59 (24.69%)	102 (42.68%)	81 (33.89%)
31–40	1 (0.26%)	101 (26.72%)	164 (43.39%)	112 (29.63%)	0 (0.00%)	5 (12.20%)	15 (36.59%)	21 (51.22%)	1 (0.13%)	165 (22.03%)	360 (48.06%)	226 (30.17%)
41–50	0 (0.00%)	55 (22.45%)	120 (48.98%)	70 (28.57%)	0 (0.00%)	14 (23.73%)	24 (40.68%)	21 (35.59%)	1 (0.22%)	104 (23.27%)	216 (48.32%)	126 (28.19%)
51–61	1 (1.18%)	23 (27.06%)	41 (48.24%)	21 (24.71%)	0 (0.00%)	8 (26.67%)	14 (46.67%)	8 (26.67%)	0 (0.00%)	24 (21.24%)	62 (54.87%)	27 (23.89%)

Results of the multivariable logistic regression analysis (Table 4) revealed that both age and BMI were independent risk factors for all three chronic diseases (all *p* < 0.05). A positive correlation was observed between age and the risk of hypertension (OR = 1.03, 95% CI: 1.02–1.04), diabetes (OR = 1.11, 95% CI: 1.08–1.13), and Dyslipidemia (OR = 1.02, 95% CI: 1.01–1.03), all *p* < 0.05. Similarly, BMI was positively associated with the risk of hypertension (OR = 1.13, 95% CI: 1.11–1.15), diabetes (OR = 1.17, 95% CI: 1.12–1.22), and Dyslipidemia (OR = 1.18, 95% CI: 1.16–1.20), all p < 0.05.

**Table 4:** Associations of age and body mass index with chronic disease risk.

Variable	Hypertension model		Diabetes model		Dyslipidemia model	
	OR (95% CI)	*p* Value	OR (95% CI)	*p* Value	OR (95% CI)	*p* Value
Main effects						
Age (years)	1.03 (1.03–1.04)	<0.001	1.11 (1.08–1.13)	<0.001	1.02 (1.01–1.03)	<0.001
BMI (kg/m^2^)	1.13 (1.11–1.15)	<0.001	1.17 (1.12–1.22)	<0.001	1.18 (1.16–1.20)	<0.001
Interaction effect						
Age × BMI	1.12 (1.05–1.20)	0.025	1.15 (1.05–1.27)	0.018	1.11 (1.03–1.20)	0.015
Model fit (Likelihood Ratio Test for interaction)		0.024		0.017		0.006

All models were adjusted for age and body mass index (BMI). The interaction term (Age × BMI) was added to assess the multiplicative interaction effect. The Likelihood Ratio Test *p*-value indicates that including the interaction term significantly improved the model fit for all three chronic diseases. OR, odds ratio; CI, confidence interval.

We further examined the multiplicative interaction between age and BMI by incorporating an interaction term into the multivariable logistic regression models (Table 4). The results demonstrated statistically significant interaction effects for all three chronic conditions: hypertension (OR = 1.12, 95% CI: 1.05–1.20, *p* = 0.025), diabetes (OR = 1.15, 95% CI: 1.05–1.27, *p* = 0.018), and Dyslipidemia (OR = 1.11, 95% CI: 1.03–1.20, *p* = 0.015). Likelihood ratio tests confirmed that models including the interaction term provided better fit than those with only main effects (all *p* < 0.05). These findings provide formal statistical evidence for a synergistic interaction between age and BMI in influencing chronic disease risk among offshore oil workers.

### Interaction effect analysis between age and BMI

**Figure 3:**
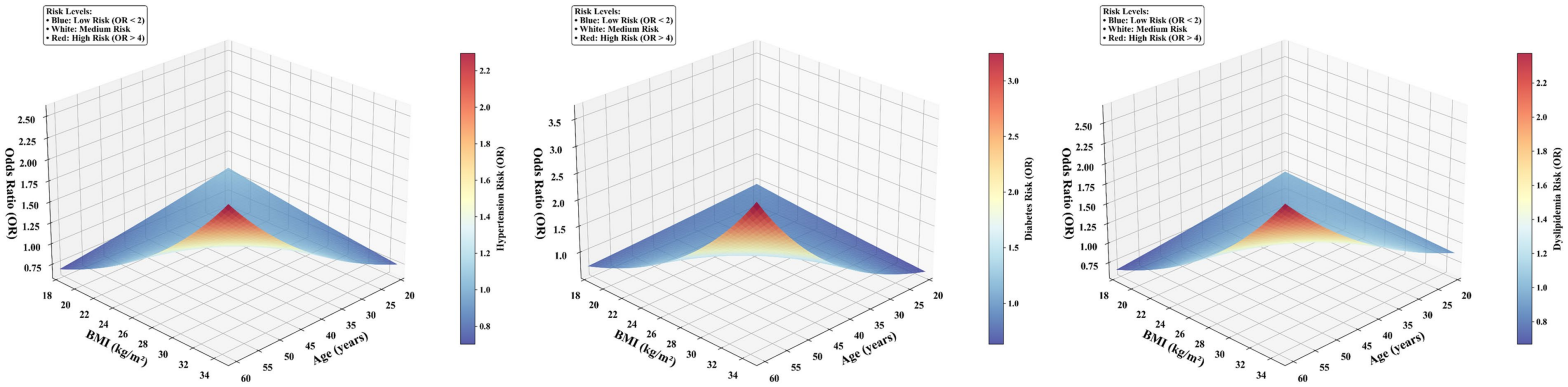
Three-dimensional plots of the interaction effect between age and body mass index (BMI) on chronic disease risk.

To further investigate the interaction effect of age and BMI on the risk of chronic diseases based on the 2024 data, a joint effect analysis was conducted by categorizing participants based on age (≤40 years vs. >40 years) and BMI (<24 kg/m^2^ vs. ≥24 kg/m^2^). The results indicated a significant interaction effect of age and BMI on the risk for all three chronic conditions (Table 5). Compared to the reference group (“≤40 years and BMI < 24 kg/m^2^”), the group “≤40 years and BMI ≥ 24 kg/m^2^” showed a marked growth risk for hypertension, diabetes, and Dyslipidemia (ORs = 1.94, 4.82, and 3.44, respectively). The group “>40 years and BMI < 24 kg/m^2^” exhibited a further increased disease risk (ORs = 1.73, 10.77, and 1.86, respectively). Notably, the group “>40 years and BMI ≥ 24 kg/m^2^” had the highest risk for chronic diseases, with the risks for hypertension, diabetes, and Dyslipidemia being 2.98, 16.11, and 4.01 times higher, respectively, than the reference group (all *p* < 0.05; Table 5). This finding indicated that the concurrent presence of older age and high BMI exerts an effect on increasing chronic disease risk that is greater than the sum of their independent effects, demonstrating a significant synergistic interaction. To visually represent this synergistic interaction, we generated three-dimensional plots based on the multivariable logistic regression models (Figure 3). These plots vividly illustrate the non-linear, accelerating increase in the risk of hypertension, diabetes, and dyslipidemia as both age and BMI increase. The highest risk strata (highlighted in red) consistently correspond to the region where age >40 years and BMI ≥ 24 kg/m^2^, providing an intuitive visual identification of the high-risk subgroup within this occupational cohort. The consistent risk patterns were further confirmed by contour plots (Supplementary Figure S1), which provide an alternative visualization of the risk gradients. To assess the temporal consistency of the age-BMI interaction effect, we conducted supplementary analyses using data from 2014 and 2019. The results from these additional years confirmed a stable and consistent pattern of the age-BMI interaction effect (see Supplementary Tables S1, S2), reinforcing the robustness of our findings.

**Table 5:** Interaction effect of age and BMI on chronic disease risk.

Group	Hypertension		Diabetes		Dyslipidemia	
	OR (95%CI)	*p* Value	OR (95%CI)	*p* Value	OR (95%CI)	*p* Value
Age ≤ 40 & BMI < 24	Reference		Reference		Reference	
Age ≤ 40 & BMI ≥ 24	1.94 (1.57–2.39)	<0.001	4.82 (2.04–11.38)	<0.001	3.44 (2.89–4.09)	<0.001
Age > 40 & BMI < 24	1.73 (1.28–2.33)	<0.001	10.77 (4.34–26.76)	<0.001	1.86 (1.44–2.39)	<0.001
Age > 40 & BMI ≥ 24	2.98 (2.37–3.74)	<0.001	16.11 (6.95–37.31)	<0.001	4.01 (3.30–4.88)	<0.001

To facilitate analysis and enhance comparability between groups, age was dichotomized into two groups using a cutoff of 40 years. Body mass index (BMI) was categorized as underweight/normal weight (<24 kg/m^2^) and overweight/obese (≥24 kg/m^2^).

Indicates significance after Bonferroni correction for multiple testing (9 tests; significance threshold set at *p* < 0.0056). All joint effect comparisons remain significant.

## Discussion

This study systematically analyzed the prevalence trends of hypertension, diabetes, and Dyslipidemia and their influencing factors among offshore oil workers in China’s Bohai Bay area from 2014 to 2024, based on health examination data. The results indicated that the prevalence of chronic diseases in this occupational population was generally at a high level, with overall mean CPR of hypertension, diabetes, and Dyslipidemia being 22.41, 2.53, and 29.64%, respectively. The ASPR of diabetes (APC = 24.08%) and Dyslipidemia (APC = 21.83%) showed a significant upward trend, whereas the trend for hypertension was not statistically significant. Age and BMI were identified as major risk factors, and a clear interaction effect between them was observed. Compared to the 2020 national data for the working-age population in China (23), which reported hypertension and Dyslipidemia prevalences of 27.50 and 8.00–18.40% respectively, the prevalence of hypertension (22.41%) and Dyslipidemia (29.64%) in this offshore worker population was higher than the national average for specific lipid disorders, with the burden of Dyslipidemia being particularly prominent.

Notably, despite the overall high burden, a marked decline in the crude prevalence of several chronic diseases was observed in 2024. Hypertension decreased from 24.38% in 2023 to 19.34%, and Dyslipidemia dropped sharply from 50.65 to 36.64%, representing the two most substantial reductions among the high-burden conditions. Diabetes, while exhibiting a lower baseline prevalence, also showed a relative decline compared to its own previous trend. The observed decline in crude prevalence rates in 2024 coincided with the phased implementation of enhanced health management policies by the CNOOC Group, which were introduced around 2023–2024 (24). These measures included a cardiovascular disease risk stratification system, individualized health improvement plans, and regular monitoring of key indicators. However, as an observational study, our findings cannot establish a causal relationship between these interventions and the decline in disease prevalence.

A critical contextual factor was the current absence of established long-term occupational population specifically targeting offshore workers. Compared with the well-known Kailuan cohort of Chinese coal miners, which also predominantly consists of middle-aged and older males (approximately 90% male, mean age 55 years), the offshore oil workers in this study exhibited a distinct chronic disease profile (25–29). The comorbidity pattern of high TG, high TC, and high BMI in the Kailuan cohort was comparable to the population in our study (25–27, 29). Our study population consisted mainly of middle-aged men averaging about 40 years old. Marked differences were observed in chronic disease prevalence: the Kailuan had higher rates of hypertension (46.83%) and dyslipidemia (38.83%), while offshore oil workers showed lower hypertension prevalence (22.41%) but considerable Dyslipidemia (29.64%) (25).

This pattern suggests that the offshore environment—particularly high-fat diets and confined spaces—may specifically affect lipid metabolism, creating a unique occupational health profile distinct from land-based miners. The distinct “dyslipidemia-predominant” profile observed in our cohort may be explained by the unique offshore environment. Emerging evidence suggests that calorie-dense diets, physical inactivity, chronic circadian disruption from shift work, and psychosocial stress in isolated settings may directly impair lipid and glucose homeostasis (7, 8, 12, 14). For instance, high-calorie diets and confined living conditions promote weight gain and insulin resistance, as reflected by elevated triglyceride-glucose indices (TyG) and C-reactive protein-triglyceride glucose (CTI) scores, which are strongly associated with cardiovascular events (12, 27, 30). Shift work and sleep deprivation further dysregulate genes involved in cholesterol synthesis and fatty acid oxidation, exacerbating dyslipidemia (4, 12). Moreover, chronic stress and isolation can activate cortisol-mediated pathways, promoting visceral adiposity and systemic inflammation (12). While cohorts like the Norwegian Offshore Petroleum Workers (NOPW) have focused primarily on cancer risks (18, 31–33), the specific cardiometabolic mechanisms in Asian offshore workers remain underexplored. Further comparison with the Norwegian Offshore Petroleum Workers (NOPW) cohort revealed similar demographic characteristics, being predominantly male (90.80%) with a comparable mean age of 42.6 years (21). However, this cohort reported substantially lower obesity rates (8% in men and 5% in women) than the 19.50% observed in our study. It was worth noting that the research focus of the NOPW cohort had been primarily on cancer, with comparatively limited emphasis on chronic diseases (32–34). This disparity suggests greater challenges in weight management and metabolic health among Chinese offshore oil workers, and highlights how health outcomes may vary across national contexts, work environments, and lifestyles within the same occupational sector.

The interaction effect of age and BMI on chronic disease risk observed in this study was consistent with findings from the Kailuan workers (23, 28, 31). Notably, BMI demonstrated a stronger association with Dyslipidemia risk (OR = 1.18) than age (OR = 1.02), establishing elevated BMI as the dominant risk factor. This pattern was particularly evident in the “Age >40 years & BMI ≥ 24 kg/m^2^” subgroup, which showed the highest diabetes risk (OR = 16.11). Importantly, even in the younger population (≤40 years), those with BMI ≥ 24 kg/m^2^ maintained growth risks for all three chronic diseases. The underlying mechanism for this age-BMI interaction likely involves dyslipidemia as a central pathway, a hypothesis strongly supported by mechanistic analyses from the Kailuan cohort. Specifically, TG and TC mediated 19.29 and 12.69%, respectively, of BMI’s effect on pancreatitis risk (20). These findings aligned with existing evidence linking obesity-related metabolic indicators to cardiovascular pathology (27, 30), collectively emphasizing weight management as the primary intervention target for chronic disease prevention in this occupational group.

This was the first study to systematically document long-term chronic disease prevalence trends among Chinese offshore oil workers, providing reliable trend analysis based on 11 years of consecutive observational data. Through the joint effect analysis of age and BMI, we identified Dyslipidemia as the predominant health concern in this population, with BMI being the primary driving factor. Comparisons with some occupational populations further clarified the distinct chronic disease profile and intervention priorities specific to this occupational group. Several limitations should be acknowledged. First, this study relied on health monitoring data from a single occupational health management system in the Bohai Bay area. As this system primarily serves the offshore oil platforms within this region, our findings are derived from a single-region sample, thus potentially limiting their generalizability to other geographic contexts. Moreover, the nature of the offshore oil workforce recorded in this system is inherently characterized by a predominance of young and middle-aged males, a demographic profile typical for this physically demanding occupation. This demographic homogeneity resulted in limited sample sizes for certain high-risk subgroups (e.g., older workers aged 51–61 years and individuals with obesity), which constrained the precision and stability of risk estimates for these specific populations. Second, the analytical scope was further constrained by the variables available from the routine health examination database. Consequently, important lifestyle confounders, such as smoking, alcohol use, diet, physical activity, and sleep patterns, were not included. Future research would benefit from actively collecting these variables through questionnaires to improve the accuracy of risk estimation.

## Conclusion

Chinese offshore oil workers face a burden of chronic diseases characterized by the rapid growth of Dyslipidemia and diabetes. The observed decline in prevalence rates in 2024, which temporally coincided with the implementation of corporate health management initiatives, is an encouraging finding that warrants further investigation to determine if a causal relationship exists. Future efforts should continue to promote comprehensive intervention strategies centered on weight management, blood lipid, and blood glucose control, integrated with dietary optimization and workplace exercise. Furthermore, health management should be deeply integrated into the production system to safeguard the health of this key occupational group and support the sustainable development of the national energy industry.

## Data Availability

The raw data supporting the conclusions of this article will be made available by the authors, without undue reservation.
