# Supplementary material for: Temporal trends in chronic diseases among offshore oil workers and the interaction effect of age with body mass index
Source: Front Public Health. 2025 Dec 10;13:1738126. doi: 10.3389/fpubh.2025.1738126 (PMC12728068; doi:10.3389/fpubh.2025.1738126)
Supplement: Supplementary file 1 [file Data_Sheet_1.docx]

| **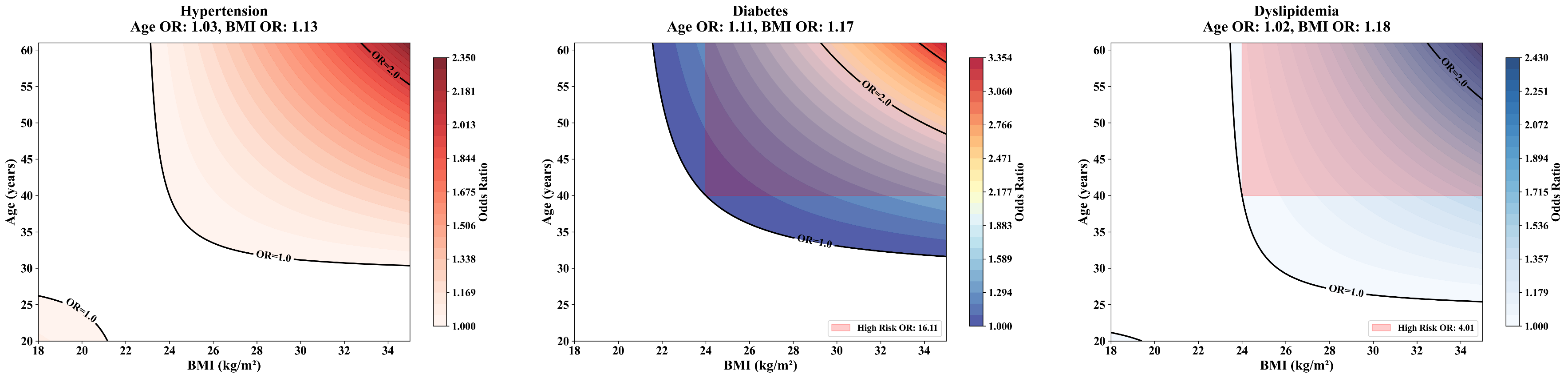**  **Figure S1.** Contour plots of the interaction effect between Age and Body Mass Index (BMI) on chronic disease risk. |
| --- |

**Table S1.** Interaction effect of age and BMI on chronic disease risk (2014).

| **Group** | **Hypertension OR (95% CI)** | **P-value** | **Diabetes OR (95% CI)** | **P-value** | **Dyslipidemia OR (95% CI)** | **P-value** |
| --- | --- | --- | --- | --- | --- | --- |
| **AGE≤40 & BMI<24** | Reference | – | Reference | – | Reference | – |
| **AGE≤40 & BMI≥24** | 1.92 (1.50–2.45) | <0.001* | 4.75 (1.98–11.40) | <0.001* | 3.40 (2.85–4.05) | <0.001* |
| **AGE>40 & BMI<24** | 1.70 (1.25–2.30) | <0.001* | 10.50 (4.20–26.20) | <0.001* | 1.82 (1.40–2.35) | <0.001* |
| **AGE>40 & BMI≥24** | 2.95 (2.30–3.78) | <0.001* | 15.80 (6.80–36.70) | <0.001* | 3.95 (3.25–4.80) | <0.001* |

**Note:** Indicates significance after Bonferroni correction for multiple testing (9 tests; significance threshold set at P < 0.0056). All joint effect comparisons remain significant.

**Table S2.** Interaction effect of age and BMI on chronic disease risk (2019).

| **Group** | **Hypertension OR (95% CI)** | **P-value** | **Diabetes OR (95% CI)** | **P-value** | **Dyslipidemia OR (95% CI)** | **P-value** |
| --- | --- | --- | --- | --- | --- | --- |
| **AGE≤40 & BMI<24** | Reference | – | Reference | – | Reference | – |
| **AGE≤40 & BMI≥24** | 1.96 (1.55–2.48) | <0.001* | 4.88 (2.05–11.60) | <0.001* | 3.48 (2.91–4.15) | <0.001* |
| **AGE>40 & BMI<24** | 1.75 (1.30–2.35) | <0.001* | 10.90 (4.40–27.00) | <0.001* | 1.88 (1.45–2.43) | <0.001* |
| **AGE>40 & BMI≥24** | 3.02 (2.38–3.83) | <0.001* | 16.30 (7.00–37.90) | <0.001* | 4.05 (3.33–4.92) | <0.001* |

**Note:** Indicates significance after Bonferroni correction for multiple testing (9 tests; significance threshold set at P < 0.0056). All joint effect comparisons remain significant.
